# The role of point of care ultrasound in prehospital critical care: a systematic review

**DOI:** 10.1186/s13049-018-0518-x

**Published:** 2018-06-26

**Authors:** Morten Thingemann Bøtker, Lars Jacobsen, Søren Steemann Rudolph, Lars Knudsen

**Affiliations:** 1 Research and Development, Prehospital Emergency Medical Services, Central Denmark Region, Aarhus, Denmark; 2 Department of Anesthesiology and Intensive Care Medicine, Aarhus University Hospital, Aarhus, Denmark; 3 Department of Research and Development, Norwegian Air Ambulance Foundation, Drøbak, Norway; 4 Air Ambulance department, Sorlandet Hospital Thrust, Arendal, Norway; 5 Centre of Head and Orthopedics, Department of Anaesthesia, Rigshospitalet, Denmark; 6 The Emergency Medical Services, Copenhagen, Denmark

**Keywords:** Prehospital, Ultrasound, Critical care, Trauma, Cardiac arrest, Dyspnea, Point of care, Education, Systematic review

## Abstract

**Background:**

In 2011, the role of Point of Care Ultrasound (POCUS) was defined as one of the top five research priorities in physician-provided prehospital critical care and future research topics were proposed; the feasibility of prehospital POCUS, changes in patient management induced by POCUS and education of providers. This systematic review aimed to assess these three topics by including studies examining all kinds of prehospital patients undergoing all kinds of prehospital POCUS examinations and studies examining any kind of POCUS education in prehospital critical care providers.

**Methods and results:**

By a systematic literature search in MEDLINE, EMBASE, and Cochrane databases, we identified and screened titles and abstracts of 3264 studies published from 2012 to 2017. Of these, 65 studies were read in full-text for assessment of eligibility and 27 studies were ultimately included and assessed for quality by SIGN-50 checklists. No studies compared patient outcome with and without prehospital POCUS. Four studies of acceptable quality demonstrated feasibility and changes in patient management in trauma. Two studies of acceptable quality demonstrated feasibility and changes in patient management in breathing difficulties. Four studies of acceptable quality demonstrated feasibility, outcome prediction and changes in patient management in cardiac arrest, but also that POCUS may prolong pauses in compressions. Two studies of acceptable quality demonstrated that short (few hours) teaching sessions are sufficient for obtaining simple interpretation skills, but not image acquisition skills. Three studies of acceptable quality demonstrated that longer one- or two-day courses including hands-on training are sufficient for learning simple, but not advanced, image acquisition skills. Three studies of acceptable quality demonstrated that systematic educational programs including supervised examinations are sufficient for learning advanced image acquisition skills in healthy volunteers, but that more than 50 clinical examinations are required for expertise in a clinical setting.

**Conclusion:**

Prehospital POCUS is feasible and changes patient management in trauma, breathing difficulties and cardiac arrest, but it is unknown if this improves outcome. Expertise in POCUS requires extensive training by a combination of theory, hands-on training and a substantial amount of clinical examinations – a large part of these needs to be supervised.

**Electronic supplementary material:**

The online version of this article (10.1186/s13049-018-0518-x) contains supplementary material, which is available to authorized users.

## Background

Prehospital Point-of-care Ultrasound (POCUS) can potentially improve patient outcome and the role of POCUS was defined as one the top five research priorities in physician-provided prehospital critical care in 2011 [1]. Three key research questions were identified; 1) which ultrasound examinations can be reliably transferred to the prehospital setting? 2) how does prehospital ultrasound affect patient management and the patient pathway? and 3) how should providers achieve and maintain specific ultrasound skills.

Although previous reviews have been positive towards the feasibility of prehospital POCUS, they were unable to demonstrate improved patient outcomes with POCUS [2, 3]. This was mainly due to very limited and heterogeneous literature of low quality lacking patient centered outcome measures. Lack of evidence of improved patient outcomes, equipment costs and training difficulties are considered significant barriers to widespread use of prehospital ultrasound [4]. Prehospital patient categories with time-critical conditions as defined by the first hour quintet may benefit from improved early diagnostics (i.e. cardiac arrest, chest pain, stroke, respiratory failure, and severe trauma) [5]. Prehospital POCUS may also alter the patient pathway for other patient groups, which may be beneficial to both the patient and the health care system.

Thus, the aim of this study was to answer the three previously defined research questions by performing a systematic review on clinical use of prehospital POCUS and on prehospital POCUS education.

## Methods

This was a commissioned systematic review on the role of POCUS in prehospital critical care conducted according to the Preferred Reporting Items for Systematic Reviews and Meta-Analyses (PRISMA) guidelines. No formal registration was performed.

### Eligibility criteria

We included studies examining all types of patients of all ages undergoing a prehospital ultrasound examination and studies examining all types of ultrasound education in all types of prehospital critical care providers. Only interventional studies (randomized and non-randomized), observational controlled and un-controlled studies and studies of diagnostic accuracy were included. Only studies published in full-text in English were included.

### Outcome measures

The primary outcome for clinical studies was patient survival within the study period. Secondary outcomes were changes in patient management, diagnostic accuracy, feasibility of the examinations and agreement between providers and experts. The primary outcome for educational studies was image acquisition skills. Secondary outcomes were image interpretation skills and theoretical knowledge.

### Information sources

As commissioned by the journal, we included studies published from January 1st, 2012. We included studies indexed in MEDLINE, EMBASE, and Cochrane Central Register of Controlled Studies. In addition, we hand-searched all included studies for references and searched the ISI Web of Science: Science Citation Index for studies citing the included studies.

### Search strategy and study selection

The search was conducted on April 24, 2017 according to the search strings supplied in the Additional file 1. Papers were imported into ENDNOTE X8 (Clarivate Analytics, Philadelphia, US) and duplicates were removed. Two reviewers (MTB and LK) independently screened papers by title and abstract and agreed on papers to assess for eligibility by their full-text version. The two reviewers then independently assessed which papers to include in the review based on their full-text. Discrepancies were solved by consensus. In case of doubt, an email was sent to the corresponding author for clarification.

### Data collection

One reviewer (MTB) extracted the following study characteristics information into a standardized spreadsheet; author last name, publication date, study type, number of participants (providers and/or patients), aim of the study, and main results. For clinical studies, type of POCUS and provider-type (physicians, paramedics, nurses etc.) was extracted. For educational studies, the educational program used was extracted.

### Assessment of quality of evidence

We used the relevant SIGN 50 checklists to assess the quality of the included studies and their risk of bias [6]. Two reviewers (SSR, LJ) independently assessed all points on the SIGN 50 checklist. When the reviewers agreed on a point, this assessment was considered final. Disagreements between reviewers were resolved by discussion using a third reviewer (MTB) as arbiter.

## Results

**Fig. 1:**
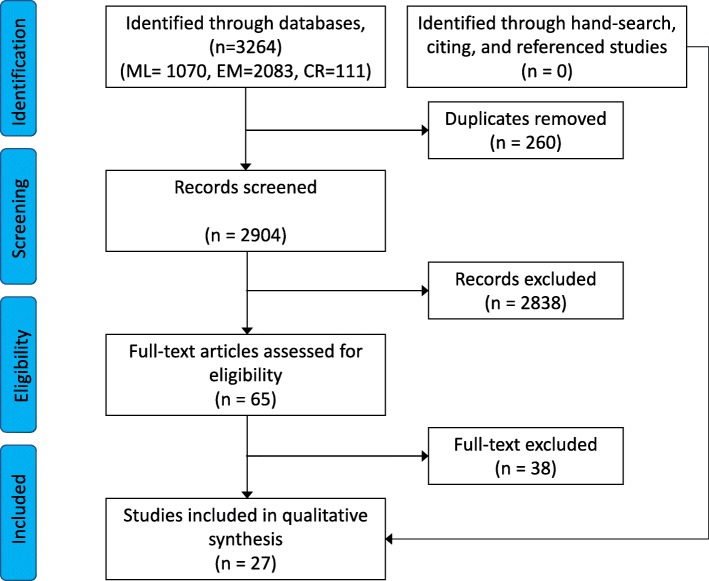
PRISMA flow diagram

We identified 3264 studies (Fig. 1). Of these, 27 studies were included in the review [7–33]. See the Additional file 1 for detailed reasons for exclusion following full-text assessment. Studies exclusively examining ultrasound in one of the first hour quintet patient groups are presented in Table 1, studies examining mixed populations or POCUS for procedural guidance in Table 2, and studies examining the effect of education in Table 3. Details on the quality of evidence assessment can be found in the Additional file 1.

**Table 1:** Included studies exclusively examining one of the first hour quintet patient groups

First author, year	n	Study type	Aim	US types, providers	Main results	Rating
Cardiac arrest only						
Aichinger, 2012	42	Prospective, observational (cohort)	To evaluate the ability of heart US to predict outcome in cardiac arrest	Heart (cardiac standstill y/n) Novice physicians	Feasibility 100%. 1/32 patients with cardiac standstill vs. 4/10 patients with cardiac movement survived to hospital discharge (*p* = 0,008). Cardiac standstill 97.1% PPV for death at scene.	+
Reed, 2017	45	Prospective, observational (cohort)	To evaluate the ability of paramedics to perform heart US during pulse check	Heart Extensively trained paramedics	Adequate view in first attempt in 80% of patients, but prolonged pauses in compressions – median 17 s (IQR 13–20).	+
Rooney, 2016	19	Cohort	To determine if paramedics could perform cardiac ultrasound in the field and correctly identify cardiac activity/standstill	Heart Novice paramedics	A total of 17/19 (89, 95% CI 67–99) exams were adequate for clinical decision-making. Correct identification of 17/17 cases of cardiac activity and 2/2 cases of cardiac standstill.	+
Chest pain						
No studies						
Suspected stroke						
Herzberg, 2014	102	Diagnostic accuracy	To evaluate the accuracy of transcranial US for neurovascular emergency diagnostics	Transcranial color-coded US in combination with clinical examination Experienced neurologists	Any stroke: sensitivity 94%, specificity 48% Major stroke: sensitivity 78%, specificity 98%	0
Breathing difficulties						
Neesse, 2012	56	Diagnostic accuracy	To evaluate the feasibility and diagnostic value of a chest ultrasound algorithm in dyspnea	Heart, anterior lungs, dorsolateral pleura Certified physicians	US helpful tool in 38/56 (68%) patients, additional therapeutic consequences drawn in 14/56 (25%). Pleural effusion found to be a 100% sensitive marker for congestive heart failure.	+
Laursen, 2016	40	Diagnostic accuracy	To assess feasibility, time-use and diagnostic accuracy of lung ultrasound for cardiogenic pulmonary edema	Anterior and lateral part of the lungs (4 regions, B-lines only) Novice physicians	Feasibility 100%. Median time used 3 min. Sensitivity 94% (CI 73–100), specificity 77% (CI 55–92), PPV 77% (CI 55–92), NPV 94% (CI 73–100)	+
Strnad, 2016	20	Prospective, observational (cohort)	To determine the usefulness of lung ultrasound in treatment monitoring with CPAP vs standard treatment in CHF	Anterior and lateral part of the lungs (15 regions), B-lines only. Physicians	Lower total number of B-lines after than before CPAP (p < 0.001). Percentage of positive US lung scans significantly reduced in several regions in the CPAP group. Changes in B-lines correlated with improved vital signs.	0
Trauma						
Brun, 2014	98	Cluster-randomized (controlled)	To compare the feasibility and efficiency of eFAST on-site, during transfer, or both	Lungs, heart, abdomen (PTX, tamponade, hemothorax, hemoperitoneum y/n) Physicians, heterogenous experience	On-site: feasibility 95.4%, efficiency 95% During transfer: feasibility 93.9%, efficiency 97% Both: feasibility 95.2%, efficiency 100% No difference between groups (w = 0.68)	–
Press, 2014	293	Diagnostic accuracy	To determine the accuracy of each component of trauma ultrasound performed by HEMS providers	Lungs, heart, abdomen (PTX, tamponade, hemothorax, hemoperitoneum y/n) Flight nurses/paramedics	Hemoperitoneum: sensitivity 46% (CI 27–94), specificity 94% (CI 89–97). Laparotomy: sensitivity 65% (CI 39–85), specificity 94%(CI 89–97). Pneumothorax: sensitivity 19% (CI 9–34), specificity 99.5% (CI 98.2–99.9). Thoracostomy: sensitivity 50% (CI 22–59), specificity 99.8% (CI 98.6–100)	+
Yates, 2017	190	Observational, controlled	To correlate prehospital trauma ultrasound findings to inhospital trauma team findings	Lungs, heart, abdomen (PTX, tamponade, hemothorax, hemoperitoneum y/n). Flight nurses/paramedics	PPV 100% NPV 98.3% Equivalent to in-hospital trauma team ultrasound	0

*Abbreviations*: *US* ultrasound, *PPV* positive predictive value, *IQR* interquartile range, *CI* confidence interval, *NPV* negative predictive value, *CPAP* continuous positive airway pressure ventilation, *PTX* pneumothorax

Rating scale: ++ High quality, + Acceptable, − Low quality/unacceptable, 0 Rejected

**Table 2:** Included studies examining mixed patient populations or ultrasound for procedural guidance

First author, year	n	Study type	Aim	US types, providers	Main results	Rating
Mixed populations						
Quick, 2016	149 patients	Controlled (prehospital paramedics vs in-hospital physicians)	To evaluate the ability of ability of in-flight thoracic US to identify pneumothorax (trauma and medical patients)	Lung (PTX), paramedics compared to ED physicians	Gold standard chest CT (*n* = 116). Prehospital sensitivity of 68% (95% CI 46–85), specificity 96% (95% CI 90–98), accuracy 91% (95% CI 85–95). Physician-based ED US; sensitivity 84% (95% CI 62–94), specificity 98% (95% CI 93–99), accuracy 96% (95% CI 90–98).	+
O’Dochertaigh, 2017	455 missions	Cohort	To describe the use of US to support interventions when used by physicians and non-physicians (trauma and medical patients)	Trauma ultrasound and IVC, highly trained physicians and non-physicians (paramedics)	Interventions was supported in US in 26% (95% CI 18–34) of cases when used by non-physicians, and in 45% (95% CI 34–56) when used by physicians (*p* < 0.006)	0
Roline, 2013	71 (41 scans)	Cohort	To evaluate the feasibility of bedside thoracic US (trauma and medical patients)	Lung (PTX), prehospital care providers (paramedics?)	In 71 eligible patients, 41 (58%) scans were completed. Level of agreement between flight crew and expert substantial with a kappa of 0.67, (95% CI 0.44–0.90). 54% of images were rated “good”. Causes for not completing US were lack of time or space limitation in aircraft.	+
Ketelaars, 2013	281 patients, 326 exams	Cohort	To evaluate the impact of US chest examinations on the care of patients in a HEMS service (trauma and cardiac arrest patients)	Heart, lung (PTX), abdomen, experienced physicians	PTX sensitivity 38%, specificity 97%, PPV 90%, NPV 69%. Treatment plan changed in 60 (21%) patients; in 10 (4%) a chest tube was abandoned; in 10 (4%) the destination for definitive care was changed, in 9 (3%) cardiopulmonary resuscitation was stopped and in 31 (11%) there were other changes.	+
Procedural guidance						
Chenaita, 2012	130 patients	Diagnostic accuracy	To estimate the diagnostic accuracy of US confirmation of gastric tube placement	Abdominal (gastric), experienced physicians	Sensitivity 98.3% (95% CI 94–99.5), specificity 100% (95% CI 75.7–100). PPV 100%, NPV 85.7%. Correlation between gastric tube size and visualization (larger tubes easier to see)	+
Brun, 2014	32	Controlled study (2-point US vs syringe test)	To estimate the diagnostic accuracy of 2-point US to confirm gastric tube placement	Esophageal, abdominal, physicians	100% visualization of gastric tube in the esophagus, 62.5% in the stomach. X-ray confirmed 28/32 in correct position. US higher diagnostic accuracy than syringe test.	0
Zadel, 2015	124 patients	Diagnostic accuracy	To assess the sensitivity and specificity of US for confirming endotracheal intubation	Lung (lung sliding and diaphragm excursion), certified physicians	Gold standard, capnography. US sensitivity 100%, specificity 100%, PPV 100%, NPV 100%. Median US time 30 s.	0

*Abbreviations*: *US* ultrasound, *PTX* pneumothorax, *CI* confidence interval, *ED* emergency department, *CT* computed tomography, *IVC* inferior vena cava, *PPV* positive predictive value, *NPV* negative predictive value

Rating scale: ++ High quality, + Acceptable, − Low quality/unacceptable, 0 Rejected

**Table 3:** Included studies examining the effect of ultrasound education

First author, year	n	Study type	Aim	Education program	Main results	Rating
Short course						
Chin, 2012	20 paramedics	Cohort	To determine if paramedics can acquire and interpret US for pneumothorax, pericardial effusion and cardiac activity	2-h session – 1 h lecture and 1 h hands-on session	After-test only: All subjects could identify the pleural line and 19/20 could obtain a cardiac view suitable for interpretation. Test score results were 9.1 out of a possible 10 (95% CI 8.6–9.6).	0
West, 2014	10 paramedics	Diagnostic accuracy	Not specified, but tested diagnostic accuracy for free fluid in abdominal trauma ultrasound	4 h course with lectures and hands-on training	Detecting of free fluid after course (peritoneal dialysis patients). Sensitivity 67%, specificity 56%. Higher false-positive rate than false-negative rate (59% vs 41%, *p* < 0.01)	0
Bhat, 2015	57 EMTs, paramedics and students	Controlled (before-and-after)	To assess the ability of EMS providers and students to accurately interpret heart and lung US images	1 h lecture on PTX, pericardial effusion and cardiac standstill	Theoretical test before and after: Test score 62.7% vs 91.1%. 95% CI for change 22–30%, p < 0.001). New post test in 19 subjects after one week: 93.1%.	+
Rooney, 2016	4 paramedics, 19 patients	Cohort	To determine if paramedics could perform cardiac ultrasound in the field and correctly identify cardiac activity/standstill	3 h course with 2 h theory and 1 h hands-on training	A total of 17/19 (89, 95% CI 67–99) exams were adequate for clinical decision-making. Correct identification of 17/17 cases of cardiac activity and 2/2 cases of cardiac standstill.	+
1- or 2-day course						
Charron, 2015	100 exams	Diagnostic accuracy	To assess the ability of emergency physicians to obtain and interpret heart and inferior vena cava views using portable US	2-day course	Parasternal short axis, long axis and subcostal views were adequate in 44, 46 and 46%, respectively. Apical 4-chamber was adequate in 67%. Agreement with experts was weak for LVF, RV size and pericardial effusion and very weak for IVC.	+
Paddock, 2015	36 paramedics, nurses and physicians	Randomized controlled study	To compare the effectiveness of training using an ultrasound simulator to traditional trauma ultrasound training	Group A: Traditional training. Group B: US simulator training. Group C: Both	No difference between groups on neither image acquisition skills nor theoretical knowledge scores.	+
Booth, 2015	11 paramedics (4 long-term)	Controlled (before-and-after)	To determine if paramedics can be trained to perform and interpret US of the heart in cardiac arrest	1-day course with 2 h theory and 4 h hands-on training.	Theoretical test before and after: Improved theoretical knowledge (test score 54% before vs 89% after, p < 0.001). Practical test only after: 88% success in image acquisition during 10-min pulse-check window. Reduced to 75% (3/4) after 10 weeks.	–
Krogh, 2016	40 physicians	Controlled (before-and-after)	To evaluate the effect of e-learning and a hands-on US course of the lungs, heart, and abdomen	1-day course with 120 min e-learning + 4 h hands-on course	Improvement in theoretical knowledge after e-learning compared to before (51.3 (SD 5.9) vs 37.5 (SD 10.0), p < 0.001). Improvement in practical US performance and image interpretation after hands-on compared to before (*p* < 0.001).	+
Longer program						
Press, 2013	33 paramedics and nurses	Controlled (before-and after)	To evaluate the effectiveness of a trauma US training curriculum and to determine if demographic factors predicted successful completion	1-day course with 2 h lectures, 4 h hands-on training + proctored session (4 exams) during 6 weeks + 60–120 min e-learning + unsupervised real-life exams	Theoretical test: none passed pre-test, 28/33 passed post-test with 78% score (*p* > 0.001 for difference). 27/33 passed structured clinical examination – only demographic factor predicting passing structured clinical exam was passing theoretical post-test.	+
Bobbia, 2015	14 physicians, 85 patients	Controlled (on experience-level)	To evaluate the interpretability of prehospital heart US based on physician experience	Experienced and non-experienced physicians defined by more or less than 50 exams after initial training (theory, 25 supervised exams)	Eight (57%) experienced physicians performed 51 (60%) exams and 6 (43%) novice physicians performed 34 (40%) exams. In multivariate analysis, only physicians experience was associated with the number of interpretable items (96% vs 56% for LVF, 98% vs 29% for PE, 92% vs 26% for RVD, and 67% vs 21% for IVC)	+
Botker, 2017	24 physicians	Controlled (before-and-after)	To evaluate the effect of a systematical education program in US of the heart and pleura on image acquisition skills, use and barriers	4 h e-learning + 1-day hands-on course + 10 supervised examinations + 3 months unsupervised exams	Proportion of images useful for interpretation increased from 0.70 (95% CI 0.65–0.75) to 0.98 (95% CI 0.95–0.99), *p* < 0.001. Used by 21/21 (100%) of prehospital providers after 4 years. Barriers for prehospital use comprised image quality in difficult patients and equipment	+

*Abbreviations*: *US* ultrasound, *CI* confidence interval, *EMT* emergency medical technician, *EMS* emergency medical services, *PTX* pneumothorax, *M-mode* motion mode, *2D-mode* 2-dimensional mode, *LVF* left ventricular function, *RV* right ventricle, *IVC* inferior vena cava, *SD* standard deviation, *PE* pericardial effusion, *RVD* right ventricular dilation

Rating scale: ++ High quality, + Acceptable, − Low quality/unacceptable, 0 Rejected

None of the included studies compared patient outcome or morbidity with and without application of POCUS.

### Cardiac arrest

Three studies that were all of acceptable quality exclusively examined ultrasound in cardiac arrest patients and demonstrated feasibility of 80–100% [7, 27, 29]. One study demonstrated a high positive predictive value of cardiac standstill for death at 97.5% when assessed by physicians [7]. POCUS performed by paramedics during pulse-checks led to prolonged pauses in compressions in another study [27]. The last study demonstrated that paramedics were able to discriminate between cardiac activity and standstill [29]. Another study of acceptable quality examined physician-based POCUS in both trauma and cardiac arrest patients and demonstrated frequent changes in patient management, among others a decision to cease resuscitation in 9 of 31 (29%) of cardiac arrest patients [18].

### Chest pain

None of the included studies specifically examined patients with chest pain.

### Stroke

One study examined transcranial ultrasound conducted by expert neurologists and demonstrated a high specificity for major stroke, but was rejected (see details of the quality of evidence assessment in the Additional file 1) [17].

### Breathing difficulties

Three studies evaluated POCUS conducted by physicians in patients with breathing difficulties [20, 21, 30]. One study of acceptable quality demonstrated 100% feasibility for simplified lung ultrasound evaluation of B-lines and a high negative predictive value of 94%, but a lower positive predictive value of 77% for congestive heart failure [20]. One study of acceptable quality demonstrated that pleural effusion is a 100% sensitive marker for congestive heart failure and that POCUS in dyspneic patients causes additional therapeutic consequences in 25% of patients [21]. The last study examining the use of B-lines by lung ultrasound to monitor the effect of treatment in heart failure patients was rejected (see details of the quality of evidence assessment in the Additional file 1) [30].

### Trauma

Three studies exclusively examined trauma patients [12, 24, 32]. One study of acceptable quality examined each component of the trauma ultrasound examination and demonstrated a positive predictive value of 90% and a negative predictive value of 98% for a required intervention due to pneumothorax, a positive predictive value of 50% with a negative predictive value of 96% for a need for laparotomy due to intraabdominal free fluid, but had an insufficient amount of pericardial effusions for reliability on this part [24]. The last two studies exclusively in trauma patients were either rejected or assessed to be of low quality (see details of the quality of evidence assessment in the Additional file 1) [12, 32]. Three studies of acceptable quality examined both trauma and medical patients and demonstrated a high level of agreement between prehospital examinations and in-hospital ultrasound assessment by expert sonographers and a change in treatment in 20% of trauma patients [18, 26, 28]. A study comparing intervention support in both trauma and medical patients when ultrasound was used by physicians and non-physicians was rejected (see details of the quality of evidence assessment in the Additional file 1) [22].

### Education

Eleven studies examined POCUS education in prehospital critical care providers [8–11, 14, 16, 19, 23, 25, 29, 31]. Three of these were either rejected or assessed to be of low quality (see details of the quality of evidence assessment in the Additional file 1) [10, 16, 31].

Two studies examining short courses were of acceptable quality [8, 29]. One demonstrated that a simple one-hour lecture improves theoretical knowledge among paramedics [8]. The other demonstrated that 2 h theory and 1 h hands-on training in paramedics with no prior ultrasound experience lead to images useful for clinical interpretation in 89% of cardiac arrest patients and correct identification of cardiac activity and cardiac standstill [29].

Three studies examining 1- or 2 day courses were of acceptable quality [14, 19, 23]. One demonstrated that theoretical knowledge, image interpretation skills and a structured observation of ultrasound examination skills in lung, heart, and abdominal ultrasound, could be improved by 2 h e-learning and 4 h hands-on course [19]. One demonstrated that after completing a two-day course, cardiac image acquisition skills were only moderate and agreement with experts was weak for left ventricular function, right ventricular size, and pericardial effusion and very weak for inferior vena cava assessment [14]. The last demonstrated that there was no difference in neither image acquisition skills nor theoretical knowledge scores when comparing traditional trauma ultrasound training to simulator-based training or both [23].

Three studies of acceptable quality examined the effect of longer educational programs [9, 11, 25]. One study examined a program comprising 1-day course with 2 h lectures and 4 h hands-on followed by at least four supervised examinations in real-life patients, 60–120 min e-learning and a number of unsupervised real-life examinations and demonstrated that 27 and 28 of 33 paramedics were able to pass a structured clinical exam and a theoretical exam, respectively [25]. Another study examined the effect of a program comprising 4 h e-learning, 1-day hands-on course, 10 supervised examinations in real-life patients and a number of unsupervised examinations and demonstrated 98% image acquisition ability after the program and that 21/21 (100%) physicians used ultrasound in the prehospital setting after the program [11]. The last study compared image acquisition skills among experienced and inexperienced physician providers (defined as more or less than 50 examinations after initial training) and demonstrated a highly significant difference for all evaluated items [9].

### Procedural guidance

Two studies evaluated the use of ultrasound to confirm gastric tube placement [13, 15]. One was rejected [13]. The other demonstrated high sensitivity and specificity of gastric ultrasound [15]. One study evaluating the effect of lung ultrasound to confirm endotracheal intubation was rejected (see details of the quality of evidence assessment in the Additional file 1) [33].

### Discussion

The main finding of this review is that considerable amounts of literature on both clinical use of prehospital POCUS and POCUS education for prehospital providers has been published since 2011, indicating a growing interest in prehospital POCUS. The most recent literature does not provide evidence of outcome improvement, but supports the use of POCUS in trauma and breathing difficulties, calls for caution in cardiac arrest, and indicates that extensive training efforts are needed for providers to obtain the necessary skills.

Previous reviews on prehospital ultrasound have pointed to a high risk of bias in the published studies and to the lack of evidence for outcome improvements [2, 3]. The authors of this review still share this concern, but consider the quality of studies included in this review as improved. Nevertheless, studies are still very heterogeneous and of variable scientific quality and the literature lacks patient centered outcome measures.

### Which ultrasound examinations can be reliably transferred to the prehospital setting?

Prehospital POCUS of the lungs for the diagnosis of pneumothorax has a moderate diagnostic accuracy and shows good agreement with experts [18, 24, 26, 28]. Positive predictive values ranges from 80 to 90% and negative predictive values from 69 to 90%. The same patterns apply to prehospital trauma ultrasound, although positive predictive value is generally lower for hemoperitoneum (around 50%) than for pneumothorax [24]. A positive POCUS finding is highly predictive of a need for intervention and seems useful for prehospital triage [18, 24]. The negative predictive values are not sufficiently high to recommend POCUS-based rule-out of serious injuries.

Prehospital POCUS of the lungs to diagnose congestive heart failure in patients with breathing difficulties displays high negative predictive value but lower positive predictive value and is reliable for rule-out, but not rule-in of congestive heart failure [20]. The addition of POCUS of the pleura may improve the positive predictive value for the diagnosis of congestive heart failure [21]. Recent studies conducted in in-hospital settings suggest that supplementing POCUS of the lungs with POCUS of the heart may further improve the positive predictive value and reduce the time to correct diagnosis [34, 35].

Prehospital POCUS of the heart is feasible and reliable for assessing simple dichotomous questions in cardiac arrest like “cardiac activity yes/no”, but may cause prolonged pauses in compressions during cardiopulmonary resuscitation [7, 27, 29]. The ability to assess more complex measures like pericardial effusion, left ventricular function, and right ventricular dilation requires extensive training and clinical ultrasound experience [9, 14]. There were no studies examining prehospital ultrasound in chest pain patients during the study period, but a recently published study demonstrated that ultrasound may also be used for early diagnosis of non-ST-elevation myocardial infarction in patients suspected of acute coronary syndrome [36].

### How does prehospital ultrasound affect patient management and the patient pathway?

Prehospital POCUS predicts the need for interventions and causes changes in patient management in both trauma, cardiac arrest, and breathing difficulties [18, 21, 24]. But, it is unknown if these changes improve patient outcomes. Since the inclusion period of this review, a secondary analysis of an included study was published [37, 22]. This study demonstrated that interventions were more likely to be supported with ultrasound in patients with markers of high acuity than in patients with presumed low-grade disease [37]. We do however question the practice of ceasing resuscitation based on cardiac standstill used in one study [18]. Early studies on this were promising [38, 39]. Yet, there are survivors following cardiac standstill in both recent and previous studies, indicating that this decision should not be based on POCUS alone [7, 40, 39].

### How should providers achieve and maintain specific ultrasound skills?

Lectures seem efficient for obtaining the simplest of image interpretation skills, while image acquisition skills require hands-on training [8, 19, 29]. The type of training used (i.e. traditional or simulation training) seems less important [23]. Systematic educational programs comprising some sort of theory (e-learning and/or lectures), hands-on training, supervised examinations, and unsupervised clinical use makes it possible to consistently produce images useful for interpretation in healthy volunteers [11, 25]. Physician experience seems to affect especially the interpretability of POCUS images of the heart after initial hands-on training and 50 examinations greatly improves image acquisition skills in real-life patients [9]. This is in accordance with a recent in-hospital study demonstrating that for most examination types, between 50 and 75 results in both excellent interpretation and good image quality in actual patients [41].

### Future research questions

Future research should address the gap in the literature demonstrating a beneficial effect of POCUS on patient centered outcome measures (improved triage, improved treatment, length-of-stay, and when possible mortality). But, to translate diagnostic accuracy into clinical utility we need to take one step back from the protocols. POCUS protocols have been defined a priori, and there is a tendency in the literature to promote specific ultrasound protocols. This is research in reverse order. When dealing with a specific patient with a specific medical history, symptoms and objective findings, some clinical questions (or differential diagnoses) arise – some of these may be answered by ultrasound. Thus, more studies on the diagnostic accuracy on specific components of a POCUS examination (such as B-lines, pleural effusion, impaired LV function) in patients with specific symptomatology (like chest pain, dyspnea, cardiac arrest, etc.) are needed to clarify which findings are key and which examinations are a waste of valuable time [42]. Only then can good controlled trials examining decision-making with and without ultrasound be planned. The Press et al. study examining sensitivity and specificity for each of the components in the trauma ultrasound examination in relation to both the relevant pathology and the associated intervention is a good example of the types of studies needed for other patient categories [24].

There is an ethical dilemma in educating prehospital critical care providers in ultrasound and randomize patients to either have the examination or not. This may be overcome by examining outcome in specific patient groups (such as abdominal aortic aneurism) in case-control studies where patients triaged directly to a specialized center by prehospital ultrasound is compared to patients admitted to local hospital and secondarily transferred, although this type of studies carries inherent risks of bias. Another way of overcoming this could be to perform cluster-randomized studies in emergency medical services where ultrasound is not already implemented. In addition, the distance to nearest hospital (and/or specialized center) may affect the value of prehospital ultrasound. Which examinations can effectively change patient management depends highly upon the local setting and organization of both prehospital and hospital care. Thus, distance and time in the emergency medical services are relevant issues for future POCUS research.

There is still a paucity of literature aiming at determining the number of examinations needed for clinical proficiency. This may be addressed by linking individual level experience to the quality of images and the correctness of clinical interpretations when compared to expert assessment.

### Limitations

Publication bias may have led to studies with neutral findings not being included – this may have been exaggerated by the choice to only include studies published in English. Especially the educational section may suffer from publication bias and conclusions must be interpreted with caution. Although the use of checklists for study quality assessment is generally recommended, the studies included in this review were very heterogeneous and we had difficulties deciding which checklists to use. Many educational studies were “before-and-after” studies. The results of this kind of study generally must be interpreted with caution due to a high risk of confounding and bias in favor of the intervention.

## Conclusion

Prehospital POCUS remains unexamined in a wide range of patient groups. Prehospital POCUS seems feasible and changes patient management in trauma and breathing difficulties. POCUS is also feasible in cardiac arrest but may cause prolonged pauses in compressions. It is unknown how prehospital POCUS affects patient outcome. The best available evidence suggests that specific POCUS skills can be achieved by a combination of theoretical education, hands-on teaching, and more than 50 clinical examinations of which a large part are supervised.

## Additional file

Additional file 1: Search strings **Table S1**. Studies excluded based on full text **Table S2**. SIGN 50 checklist of cohort studies included in the review **Table S3**. SIGN 50 checklist of controlled studies included in the review 5 **Table S4**. SIGN 50 checklist of diagnostic accuracy studies included in the review references. (PDF 229 kb)

## Abbreviations

POCUS: Point of Care Ultrasound
PRISMA: Preferred Reporting Items for Systematic Reviews and Meta-Analyses
