# Supplementary material for: The role of point of care ultrasound in prehospital critical care: a systematic review
Source: Scand J Trauma Resusc Emerg Med. 2018 Jun 26;26:51. doi: 10.1186/s13049-018-0518-x (PMC6019293; doi:10.1186/s13049-018-0518-x)
Supplement: Supplementary file 1 — Search strings Table S1. Studies excluded based on full text Table S2. SIGN 50 checklist of cohort studies included in the review Table S3. SIGN 50 checklist of controlled studies included in the review 5 Table S4. SIGN 50 checklist of diagnostic accuracy studies included in the review references. (PDF 229 kb) [file 13049_2018_518_MOESM1_ESM.pdf]

Supplement to: The role of ultrasound in physician-provided prehospital critical care: a systematic review

Morten Thingemann Bøtker, Søren Steemann Rudolph, Lars Jacobsen, Lars Knudsen

Content

Search strings	2
Supplementary Table 1. Studies excluded based on full-text	3
Supplementary Table 2. SIGN 50 checklist of cohort studies included in the review	4
Supplementary Table 3. SIGN 50 checklist of controlled studies included in the review	5
Supplementary Table 4. SIGN 50 checklist of diagnostic accuracy studies included in the review	6
Supplementary references	7

Search Strings

Pubmed
("Emergency Medical Services"[Mesh] OR "Emergency Medical Services" OR prehospital OR pre-hospital OR out-of-hospital OR "out of hospital") AND ([ultraso*[tiab] OR sonograph*[tiab] OR echocardiograph*[tiab])
Limited to 2012-2017

Cochrane
#1 MeSH descriptor: [Emergency Medical Services] explode all trees
#2 "Emergency Medical Services"
#3 prehospital OR pre-hospital OR out-of-hospital OR "out-of-hospital"
#4 ultraso* or sonograph* or echocardiograph*
(#1 OR #2 OR #3) AND #4
Limited to 2012-2017

EMBASE:
#1 'emergency health service'/exp OR 'emergency care'/exp OR prehospital OR 'pre-hospital' OR 'out-of-hospital'
#2 'ultraso*' OR 'sonograph*' OR 'echocardiograph*'
#1 AND #2
Limited to 2012-2017

Supplementary Table 1. Studies excluded based on full-text

Number	Study	Cause
1	Brun et al., 2013. The value of prehospital echocardiography in shock management[1]	Case report
2	Callerova et al, 2015. TRACE: A new protocol for ultrasound examination during out-of-hospital cardiac arrest[2]	Conference abstract
3	Castellani et al., 2012. Telementoring: Ultrasound training and research[3]	Conference abstract
4	Grmec et al., 2012. Sonographic B-Line, NT-proBNP and pressure of end-tidal CO2 (petCO2) in diagnosis of acute heart failure in prehospital emergency setting[4]	Conference abstract
5	Hanlin et al., 2016. Airway ultrasound for the confirmation of endotracheal tube placement in military flight medic trainees[5]	Conference abstract
6	Jones et al., 2014. The effect of thoracic ultrasound for the detection of pneumothorax on medical decisionmaking in trauma patients in the out-of-hospital setting[6]	Conference abstract
7	Kamp et al., 2012. Ultrasound enhanced prehospital thrombolysis using microbubbles in patients with acute ST elevation myocardial infarction[7]	Conference abstract
8	Knapp et al., 2015. Quality and accuracy of fast exams performed by ems providers in the outof-hospital setting[8]	Conference abstract
9	Knap et et al., 2012. Emergency medical services focused assessment with sonography in trauma and cardiac ultrasound in cardiac arrest: The training phase[9]	Conference asbstract
10	Lahham et al., 2015. Prehospital assessment with ultrasound in emergencies-pause II[10]	Conference abstract
11	Lema et al., 2014. Ultrasound identification of successful endotracheal tube placement by paramedics and residents[11]	Conference abstract
12	Lyon et al., 2012 Is there a role for pre-hospital chest ultrasound in trauma patients?[12]	Conference abstract
13	Ogedegbe et al., 2012. A novel portable telesonography system for out-of-hospital trauma care[13]	Conference abstract
14	Schlachetzki et al., 2014. Pre-hospital thrombolysis - Is the ultrasound diagnosis of occlusion enough?[14]	Conference abstract
15	Skulec et al., 2015. TRACE: A new protocol for ultrasound examination during out-of-hospital cardiac arrest[15]	Conference abstract
16	Vitto et al., 2015. Implementation of a flight medical crew ultrasound training program[16]	Conference abstract
17	Schlachetzki et al., 2012. Transcranial ultrasound from diagnosis to early stroke treatment: part 2: prehospital neurosonography in patients with acute stroke: the Regensburg stroke mobile project[17]	Cohort included in other publication with more clinical data (Herzberg et al., 2014)
18	Atkinsion et al., 2014. Coming of age: emergency point of care ultrasonography in Canada[18]	Editorial
19	Lien, 2014. Emergency ultrasound[19]	Editorial
20	Brun et al., 2014. Ultrasound evaluation of the nasogastric tube position in prehospital[20]	Non-english
21	Weilbach et al., 2017. Introduction of Prehospital Emergency Ultrasound into an Emergency Medical Service Area[21]	Non-english
22	Brandt et al., 2016. The use of ultrasound to identify veins for peripheral venous access in morbidly obese patients[22]	Not prehospital
23	Breitkreutz et al., 2013. Thorax, trachea, and lung ultrasonography in emergency and critical care medicine: Assessment of an objective structured training concept[23]	Not prehospital
24	Cazes et al., 2013. Emergency ultrasound: A prospective study on sufficient adequate training for military doctors[24]	Not prehospital
25	Chaudery et al., 2016. Can contrast-enhanced ultrasonography improve Zone III REBOA placement for prehospital care?[25]	Not prehospital
26	Jang et al., 2012. Sonographic assessment of jugular venous distension and B-type natriuretic peptide levels in patients with dyspnoea[26]	Not prehospital
27	Jang et al., 2012. The technical errors of physicians learning to perform focused assessment with sonography in trauma[27]	Not prehospital
28	Kim et al., 2016. Can serial focussed echocardiographic evaluation in life support (FEEL) predict resuscitation outcome or termination of resuscitation (TOR)? A pilot study[28]	Not prehospital
29	Lee et al., 2015. Combined ECG, echocardiographic, and biomarker criteria for diagnosing acute myocardial infarction in out-of-hospital cardiac arrest patients[29]	Not prehospital
30	Ozkan et al., 2015. Stethoscope versus point-of-care ultrasound in the differential diagnosis of dyspnea: a randomized trial[30]	Not prehospital
31	Slikkerveer et al., 2012. Ultrasound enhanced prehospital thrombolysis using microbubbles infusion in patients with acute ST elevation myocardial infarction: pilot of the Sonolysis study[31]	Not prehospital
32	Ogedegbe et at., 2012. Development and evaluation of a novel, real time mobile telesonography system in management of patients with abdominal trauma: Study protocol[32]	Study protocol
33	Hong et al., 2012. Detecting prehospital hemoperitoneum remotely through fast and 3 g network: A simulation study[33]	Simulation study, no patients included
34	Kirkpatrick et al., 2016. Remote just-in-time telementored trauma ultrasound: a double-factorial randomized controlled trial examining fluid detection and remote knobology control through an ultrasound graphic user interface display[34]	Simulation study, no patients included
35	Lyon et al., 2012. M-mode ultrasound for the detection of pneumothorax during helicopter transport[35]	Simulation study, no patients included
36	Lyon et al., 2012. Ultrasound detection of the sliding lung sign by prehospital critical care providers[36]	Simulation study, no patients included
37	McBeth et al., 2013. Help is in your pocket: the potential accuracy of smartphone- and laptop-based remotely guided resuscitative telesonography[37]	Simulation study, no patients included
38	Song et al., 2013. Clinical applicability of real-time, prehospital image transmission for FAST (Focused Assessment with Sonography for Trauma)[38]	Simulation study, no patients included

Supplementary Table 2. SIGN 50 checklist of cohort studies included in the review

		In a well conducted cohort study….	Aichinger, 2012	Chin, 2013	Ketelaars, 2013	ODochartaigh, 2017	Reed, 2017	Roline, 2013	Rooney, 2016	Strnad, 2016
			**Study**							
**Internal Validity**	1.1	The study addresses an appropriate and clearly focused question	Yes	Yes	Yes	Yes	Yes	Yes	Yes	Yes
	1.2	The two groups being studied are selected from source populations that are comparable in all respects other that the factor under investigation	Not reported	Not reported	Not reported	No	Not reported	Not reported	Not reported	Yes
	1.3	The study indicates how many of the people asked to take part did so, in each of the groups being studied	Not reported	No	Not reported	Yes	Yes	No	No	No
	1.4	The likelihood that some eligible subjects might have the outcome at the time of enrollment is assessed and taken into account in the analysis	Not reported	Yes	Not reported	Not reported	Not reported	Can't say	Yes	Not reported
	1.5	What percentage of individuals or clusters recruited into each arm of the study dropped out before the study was completed	Total 0	Total 0	Not reported	Not reported	Total 13	Total 0	Total 80	I/C 14/0
	1.6	Comparison is made between full participants and those lost to follow up, by exposure status	Not reported	Not reported	No	Not reported	Not reported	Not reported	No	Not reported
	1.7	The outcomes are clearly defined	Yes	Yes	Yes	Yes	Yes	Yes	Yes	Yes
	1.8	The assessment of outcome is made blind to exposure status	No	Not reported	Not reported	Not reported	Yes	Yes	Not reported	No
	1.9	Where blinding not possible, there is some recognition that knowledge of exposure status could have influenced the assessment of outcome	Can't say	Can't say	Can't say	Can't say	Can't say	Can't say	Can't say	Yes
	1.10	The measure of assessment of exposure is reliable	Yes	Yes	Can't say	Yes	Yes	Yes	Can't say	Yes
	1.11	Evidence of other sources is used to demonstrate that the method of outcome assessment is valid and reliable	Yes	Yes	Not reported	Can't say	Yes	No	Yes	Yes
	1.12	Exposure level or prognostic factor is assessed more than once	Yes	No	Not reported	No	Not reported	No	No	No
	1.13	The main potential confounders are identified and taken into account in the design and analysis	Yes	No	No	No	No	No	No	Yes
	1.14	Confidence intervals are provided	No	No	No	Yes	No	Yes	Yes	No
**Overall**	2.1	How well was the study done to minimise the risk of bias or confounding?	+	0	+	0	+	+	+	0
	2.2	There is a clear evidence of an association between exposure and outcome	Yes	No	Can't say	No	Yes	Yes	Yes	No
	2.3	Are the results of this study directly applicable to the patient group targeted?	Yes	Yes	Yes	Yes	Yes	Yes	Yes	Yes

Legend

Yes	green circle
Can't say	yellow circle
No	red circle
Not reported	blue circle

Quality rating

High quality	++
Acceptable	+
Unacceptable	0

Does not apply	Does not apply (symbol)

Supplementary Table 3. SIGN 50 checklist of controlled studies included in the review

		In a well conducted controlled study….	Bobbia, 2015	Botker, 2017	Brun, 2014	Krogh, 2016	Bhat, 2015	Booth, 2015	Brun, 2014	Paddock, 2015	Press, 2013	Quick, 2016	Yates, 2017
			**Study**										
Internal Validity	1.1	The study addresses an appropriate and clearly focused question	Yes	Yes	Yes	Yes	Yes	Yes	Yes	Yes	Yes	Yes	Yes
	1.2	The assignment of subjects to treatment groups is randomized	No	No	Yes	No	No	No	No	Yes	No	No	No
	1.3	*An adequate concealment method is used*	Does not apply	Does not apply	Can't say	Does not apply	Does not apply	Does not apply	Does not apply	Can't say	Does not apply	Does not apply	Does not apply
	1.4	The design keeps subjects and investigators 'blind' about treatment allocation	Does not apply	Does not apply	Can't say	Does not apply	Does not apply	Does not apply	Does not apply	Yes	Does not apply	Does not apply	Does not apply
	1.5	The treatment and control groups are similar at the start of the trial	No	No	Can't say	No	No	Can't say	Can't say	Yes	Can't say	Can't say	No
	1.6	The only difference between groups is the treatment under investigation	No	Can't say	Can't say	Can't say	Can't say	Can't say	Can't say	No	Can't say	Can't say	Can't say
	1.7	All relevant outcomes are measured in a standard, valid and reliable way	Yes	Yes	No	Yes	Can't say	Yes	No	Yes	Yes	Yes	No
	1.8	What percentage of the individuals or clusters recruited into each treatment arm of the study dropped out before the study was completed?	0%	Total 10%	Can't say	Total 34%	Total 2%	Total 9%	Can't say	0%	Total 3%	Total 22%	Total 48%
	1.9	*All the subjects are analyzed in the groups to which they were randomly allocated (often referred to as intention to treat analysis)*	Does not apply	Does not apply	Does not apply	Does not apply	Does not apply	Does not apply	Does not apply	Yes	Does not apply	Does not apply	No
	1.10	Where the study is carried out at more than one site, results are comparable for all sites	Does not apply	Does not apply	Does not apply	Does not apply	Yes	Does not apply	Does not apply	Does not apply	Does not apply	Does not apply	Does not apply
Overall	2.1	How well was the study done to minimize bias?	+	+	-	+	+	-	0	+	+	+	-
	2.2	It seems certain that the overall effect is due to the study association	Yes	Yes	No	Yes	Yes	Yes	Yes	Yes	Yes	Yes	No
	2.3	The results are directly applicable to the patients targeted	Yes	Yes	No	Yes	Yes	Yes	No	Yes	Yes	Yes	No

Legend

Yes	(green circle)
Can't say	(yellow circle)
No	(red circle)
Not reported	(light blue circle)
Does not apply	(blue circle)

Quality rating

High quality	++
Acceptable	+
Low quality	-
Unacceptable	0

Supplementary Table 4. SIGN 50 checklist of diagnostic accuracy studies included in the review

		In a well conducted diagnostic accuracy study….	Herzberg, 2014	Laursen, 2016	Charron, 2015	Chenaita, 2012	Neesse, 2012	Zadel, 2015	Press, 2014	West, 2014
**Patient selection**	1.1	A consecutive sequence or random selection of patients is enrolled	Yes	No	No	Can't say	Yes	Can't say	No	Yes
	1.2	Case – control methods are not used	Yes	Yes	Yes	Yes	Yes	Yes	Yes	Yes
	1.3	Inappropriate exclusions are avoided	No	Can't say	Can't say	Can't say	Can't say	No	No	Yes
	1.4	The included patients and settings match the key question	Yes	Yes	Yes	Yes	Yes	Yes	Yes	No
**Index test**	2.1	The index test results are interpreted without knowledge of the results of the reference standard	Yes	Yes	Yes	Yes	Yes	No	Yes	Can't say
	2.2	If a threshold is used, it is pre-specified	Yes	Yes	Can't say	No	Can't say	Can't say	Yes	Can't say
	2.3	The index test, its conduct, and its interpretation is similar to that used in practice with the target population of the guideline	Yes	Yes	Can't say	Yes	Can't say	Yes	Yes	Can't say
**Reference standard**	3.1	The reference standard is likely to correctly identify the target condition	Yes	Yes	Yes	Yes	Yes	Yes	Yes	Yes
	3.2	Reference standard results are interpreted without knowledge of the results of the index test	Can't say	Yes	Can't say	Can't say	Yes	No	Yes	Can't say
	3.3	The target condition as defined by the reference standard matches that found in the target population of the guideline	Yes	Yes	Yes	Yes	Yes	Yes	Yes	Can't say
**Flow & timing**	4.1	There is an appropriate interval between the index test and reference standard	Yes	Yes	Yes	Can't say	Yes	Yes	Yes	Can't say
	4.2	All patients receive the same reference standard	No	Yes	Yes	Yes	No	Yes	No	Yes
	4.3	All patients recruited into the study are included in the analysis	No	Yes	Yes	Yes	Yes	Yes	Can't say	No
**Overall**	2.1	How well was the study done to minimize bias?	0	+	+	+	+	0	+	0
	2.2	What is the applicability of this study to the target population	Some indirectness	Directly applicable	Directly applicable	Directly applicable	Directly applicable	Some indirectness	Directly applicable	Some indirectness

Legend

Yes	green circle
Can't say	yellow circle
No	red circle
Not reported	light blue circle
Does not apply	blue circle

Quality rating

High quality	++
Acceptable	+
Unacceptable	0

Applicability

Directly applicable	green circle
Some indirectness	yellow circle

Supplementary references

1. Brun PM, Chenaitia H, Gonzva J, Bessereau J, Bobbia X, Peyrol M. The value of prehospital echocardiography in shock management. Am J Emerg Med. 2013;31:442.
2. Callerova J, Skulec R, Knor J, Cerny V. TRACE: A new protocol for ultrasound examination during out-ofhospital cardiac arrest. Crit Care. 2015; DOI:10.1186/cc14496.
3. Castellani S, Meneghetti G. Telementoring: Ultrasound training and research. Cerebrovasc Dis. 2012; DOI:10.1159/000338951.
4. Grmec S, Hajdinjak E, Prosen G. Sonographic B-Line, NT-proBNP and pressure of end-tidal CO2 (petCO2) in diagnosis of acute heart failure in prehospital emergency setting. Circulation. 2012; DOI: 10.1161/CIR.0b013e31824fcd6b.
5. Hanlin E. Airway ultrasound for the confirmation of endotracheal tube placement in military flight medic trainees. Acad Emerg Med. 2016; DOI:10.1111/acem.12974.
6. Jones RA, Tabbut M, Emerman C, Stout S. The effect of thoracic ultrasound for the detection of pneumothorax on medical decisionmaking in trauma patients in the out-of-hospital setting. Ann Emerg Med. 2014; DOI:10.1016/j.annemergmed.2014.07.109.
7. Kamp O, Slikkerveer J. Ultrasound enhanced prehospital thrombolysis using microbubbles in patients with acute ST elevation myocardial infarction. Circulation. 2012; doi:10.1161/CIR.0b013e31824fcd6b.
8. Knapp B, Byars D, Ford L. Quality and accuracy of fast exams performed by ems providers in the outof-hospital setting. Acad Emerg Med. 2015; doi:10.1111/acem.12644.
9. Knapp B, Byars D, Stewart V, Ryszkiewicz R, Evans D. Emergency medical services focused assessment with sonography in trauma and cardiac ultrasound in cardiac arrest: The training phase. Acad Emerg Med. 2012; DOI:10.1111/j.1553-2712.2012.01332.x.
10. Lahham S, Rooney K, Sloane B, Fox JC. Prehospital assessment with ultrasound in emergencies-pause II. Acad Emerg Med. 2015; DOI:10.1111/acem.12644.
11. Lema PC, Wilson J, O'Brien M, Lindstrom H, Tanski C, Consiglio J et al. Ultrasound identification of successful endotracheal tube placement by paramedics and residents. Ann Emerg Med. 2014; DOI:10.1016/j.annemergmed.2014.07.043.
12. Lyon R, Weaver A, Wise D, Davies G, Lockey D. Is there a role for pre-hospital chest ultrasound in trauma patients? Resuscitation. 2012; DOI:org/10.1016/j.resuscitation.2012.08.218.
13. Ogedegbe C. A novel portable telesonography system for out-of-hospital trauma care. Ann Emerg Med. 2012; DOI:10.1016/j.annemergmed.2012.06.236.
14. Schlachetzki F, Herzberg M, Pflug K, Backhaus R, Baldaranov D, Ertl M et al. Pre-hospital thrombolysis - Is the ultrasound diagnosis of occlusion enough? Cerebrovasc Dis. 2014; DOI: 10.1159/000363090.
15. Skulec R, Truhlar A, Knor J, Cerny V. TRACE: A new protocol for ultrasound examination during out-of-hospital cardiac arrest. Resuscitation. 2015; DOI: 10.1016/j.resuscitation.2015.09.111.
16. Vitto MJ, Spector D, Evans DP. Implementation of a flight medical crew ultrasound training program. Academic Emergency Medicine. 2015; DOI:10.1111/acem.12644.
17. Schlachetzki F, Herzberg M, Holscher T, Ertl M, Zimmermann M, Ittner KP et al. Transcranial ultrasound from diagnosis to early stroke treatment: part 2: prehospital neurosonography in patients with acute stroke: the Regensburg stroke mobile project. Cerebrovasc Dis. 2012;33:262-71.
18. Atkinson P, Ross P, Henneberry R. Coming of age: emergency point of care ultrasonography in Canada. CJEM. 2014;16:265-8.
19. Lien WC. Emergency ultrasound. J Med Ultrasound. 2014; DOI:10.1016/j.jmu.2014.05.007.
20. Brun PM, Chenaitia H, Bessereau J, Leyral J, Barberis C, Pradel-Thierry AL et al. Ultrasound evaluation of the nasogastric tube position in prehospital. Ann Fr Anest Reanim. 2012;31:416-20.
21. Weilbach C, Kobiella A, Rahe-Meyer N, Johanning K. [Introduction of Prehospital Emergency Ultrasound into an Emergency Medical Service Area]. Anaesthesist. 2017;66:21-7.
22. Brandt HGS, Jepsen CH, Hendriksen OM, Lindekær A, Skjønnemand M. The use of ultrasound to identify veins for peripheral venous access in morbidly obese patients. Dan Med J. 2016;63.
23. Breitkreutz R, Dutiné M, Scheiermann P, Hempel D, Kujumdshiev S, Ackermann H et al. Thorax, trachea, and lung ultrasonography in emergency and critical care medicine: Assessment of an objective structured training concept. Emerg Med Int. 2013; DOI:10.1155/2013/312758.
24. Cazes N, Desmots F, Geffroy Y, Renard A, Leyral J, Chaumoître K. Emergency ultrasound: A prospective study on sufficient adequate training for military doctors. Diagn Interv Imaging. 2013;94:1109-15.
25. Chaudery M, Clark J, Morrison JJ, Wilson MH, Bew D, Darzi A. Can contrast-enhanced ultrasonography improve Zone III REBOA placement for prehospital care? Journal Trauma Acute Care Surg. 2016; doi:10.1097/TA.0000000000000863.
26. Jang T, Aubin C, Naunheim R, Lewis LM, Kaji AH. Sonographic assessment of jugular venous distension and B-type natriuretic peptide levels in patients with dyspnoea. Emerg Med J. 2012;29:477-81.

27. Jang T, Kryder G, Sineff S, Naunheim R, Aubin C, Kaji AH. The technical errors of physicians learning to perform focused assessment with sonography in trauma. Acad Emerg Med. 2012;19:98-101.
28. Kim HB, Suh JY, Choi JH, Cho YS. Can serial focussed echocardiographic evaluation in life support (FEEL) predict resuscitation outcome or termination of resuscitation (TOR)? A pilot study. Resuscitation. 2016;101:21-6.
29. Lee SE, Uhm JS, Kim JY, Pak HN, Lee MH, Joung B. Combined ECG, echocardiographic, and biomarker criteria for diagnosing acute myocardial infarction in out-of-hospital cardiac arrest patients. Yonsei Med J. 2015;56:887-94.
30. Ozkan B, Unluer EE, Akyol PY, Karagoz A, Bayata MS, Akoglu H et al. Stethoscope versus point-of-care ultrasound in the differential diagnosis of dyspnea: a randomized trial. Eur J Emerg Med. 2015;22:440-3.
31. Slikkerveer J, Kleijn SA, Appelman Y, Porter TR, Veen G, Rossum AC et al. Ultrasound enhanced prehospital thrombolysis using microbubbles infusion in patients with acute ST elevation myocardial infarction: pilot of the Sonolysis study. Ultrasound Med Biol. 2012; doi:10.1016/j.ultrasmedbio.2011.11.001.
32. Ogedegbe C, Morchel H, Hazelwood V, Chaplin WF, Feldman J. Development and evaluation of a novel, real time mobile telesonography system in management of patients with abdominal trauma: Study protocol. BMC Emerg Med. 2012; DOI: 10.1186/1471-227X-12-19.
33. Hong KJ, Song KJ, Song SW, Shin SD. Detecting prehospital hemoperitoneum remotely through fast and 3 g network: A simulation study. J Emerg Med. 2012; DOI: 10.1016/j.jemermed.2012.09.066.
34. Kirkpatrick AW, McKee I, McKee JL, Ma I, McBeth PB, Roberts DJ et al. Remote just-in-time telementored trauma ultrasound: a double-factorial randomized controlled trial examining fluid detection and remote knobology control through an ultrasound graphic user interface display. Am J Surg. 2016; 211:894-902.
35. Lyon M, Shiver SA, Walton P. M-mode ultrasound for the detection of pneumothorax during helicopter transport. Am J Emerg Med. 2012;30:1577-80.
36. Lyon M, Walton P, Bhalla V, Shiver SA. Ultrasound detection of the sliding lung sign by prehospital critical care providers. Am J Emerg Med. 2012;30:485-8.
37. McBeth P, Crawford I, Tiruta C, Xiao Z, Zhu GQ, Shuster M et al. Help is in your pocket: the potential accuracy of smartphone- and laptop-based remotely guided resuscitative telesonography. Telemed J E Health. 2013;19:924-30.
38. Song KJ, Shin SD, Hong KJ, Cheon KW, Shin I, Song SW et al. Clinical applicability of real-time, prehospital image transmission for FAST (Focused Assessment with Sonography for Trauma). J Telemed Telecare. 2013;19:450-5.
